# Progress on pivotal role and application of exosome in lung cancer carcinogenesis, diagnosis, therapy and prognosis

**DOI:** 10.1186/s12943-021-01312-y

**Published:** 2021-01-27

**Authors:** Ming-Yue Li, Li-Zhong Liu, Ming Dong

**Affiliations:** 1 Biomedical Equipment Department, Bioland Laboratory (Guangzhou Regenerative Medicine and Health Guangdong Laboratory), Building 3, No.188, KaiYuan Road, Huangpu District, Guangzhou, Guangdong China; 2 Department of Physiology, School of Medicine, Shenzhen University Health Science Center, Shenzhen University, A7-304, Shenzhen University Xili Campus, Nanshan District, Shenzhen, 518055 China

**Keywords:** Lung cancer cell-derived exosomes (LCCDEs), carcinogenesis, diagnosis, therapy, prognosis

## Abstract

Lung cancer is often diagnosed at an advanced stage and has a poor prognosis. Conventional treatments are not effective for metastatic lung cancer therapy. Although some of molecular targets have been identified with favorable response, those targets cannot be exploited due to the lack of suitable drug carriers. Lung cancer cell-derived exosomes (LCCDEs) receive recent interest in its role in carcinogenesis, diagnosis, therapy, and prognosis of lung cancer due to its biological functions and natural ability to carry donor cell biomolecules. LCCDEs can promote cell proliferation and metastasis, affect angiogenesis, modulate antitumor immune responses during lung cancer carcinogenesis, regulate drug resistance in lung cancer therapy, and be now considered an important component in liquid biopsy assessments for detecting lung cancer. Therapeutic deliverable exosomes are emerging as promising drug delivery agents specifically to tumor high precision medicine because of their natural intercellular communication role, excellent biocompatibility, low immunogenicity, low toxicity, long blood circulation ability, biodegradable characteristics, and their ability to cross various biological barriers. Several studies are currently underway to develop novel diagnostic and prognostic modalities using LCCDEs, and to develop methods of exploiting exosomes for use as efficient drug delivery vehicles. Current status of lung cancer and extensive applicability of LCCDEs are illustrated in this review. The promising data and technologies indicate that the approach on LCCDEs implies the potential application of LCCDEs to clinical management of lung cancer patients.

## Background

Lung cancer is the most frequent cancer and leading cause of cancer death worldwide [1]. Small-cell lung carcinoma (SCLC) and non-small cell lung carcinoma (NSCLC), accounting for 15% and 85% of all lung cancers respectively, are two main subtypes [2]. SCLC is highly related to smoking. NSCLC is further classified into three main types: squamous cell carcinoma (SCC), adenocarcinoma (AD), and large cell carcinoma (LCC) [2]. SCC and LCC, comprising 25–30% and 5–10% of all lung cancer cases respectively, are strongly correlated with cigarette smoking [3, 4]. AD is the most common type both in smokers and nonsmokers and comprises 40-45% of all lung cancer cases [5]. Tumor stage is important for treatment and prognosis. The 5-year survival of lung cancer is about 19% [6]. Low lung cancer survival rate is due to more than 50% NSCLC patients diagnosed with metastatic disease [7]. Patients with early-stage lung cancer have better prognosis than those with more advanced stage. 5-year relative survival rate reaches 90% for stage 1A1 but drops below 10% for stage 4 NSCLC. Among patients with SCLC, 5-year relative survival rates are about 30% for limited disease and below 10% for extensive disease [8]. Because of its extraordinary disease burden and international variability in trends of population growth, aging, and smoking behaviors, the global lung cancer epidemiology requires continual monitoring [9].

All cells release extracellular vesicles, which are broadly divided into two categories of ectosomes and exosomes, as part of their normal or abnormal physiology [10]. Exosomes are endosomal origin and nano-sized vesicles with a size range of ~40-160 nm (average ~100 nm) in diameter [10]. One speculated role of exosomes is to remove excess and/or unnecessary constituents from cells to maintain cellular homeostasis [10]. When exosomes are taken up by other cells, the cargoes are transferred and influence the phenotype of recipient cells, indicating exosomes as essential mediators of cell-cell communication [11]. Current status of lung cancer and extensive applicability of Lung cancer cell-derived exosomes (LCCDEs) are illustrated in this review. LCCDEs participate in lung cancer carcinogenesis and drug resistance. Functional study of LCCDEs is useful to elucidate the pathogenesis of lung cancer. LCCDEs contain potential diagnostic and prognostic biomarkers, which can be developed for liquid biopsy of lung cancer. Delivery of therapeutic agents by engineering LCCDEs is a novel approach for lung cancer precision and personalized therapy.

### Risk factors for lung cancer

Risk factors for developing lung cancer have been identified, with cigarette smoking being a major factor along with other environmental, chronic disease, and genetic risk factors. Since about 80% of lung cancers develop in current or former tobacco smokers and up to 20% of all cancer deaths worldwide could be prevented by tobacco smoking elimination, cigarette smoking outweighs all other factors [8]. At least 50 carcinogens has been identified in tobacco smoke, of which, tobacco-specific N-nitrosamines formed by nicotine nitrosation are particularly concerned [12]. And tobacco-specific N-nitrosamine 4-(methylnitrosamino)-1(3-pyridyl)-1-butanone (NNK) seems to be the most important lung cancer inducer [13]. Global statistics indicates that lung cancer in never smokers (LCNS) accounts for about 20% of lung cancer [14]. At least 17% of LCNS are attributable to exposure to high levels of environmental tobacco smoke (ETS) or secondhand smoke [15]. 24% excess risk for lung cancer is found in nonsmokers who lives with a smoker [16].

The incidence of lung cancer without tobacco smoking history is increasing [17]. The polluted air, especially particulate matter 2.5 (PM_2.5_) has been indicated as the major factor for LCNS incidence [18]. Air pollution has been a major environmental problem for more than two decades and is worsening in many large populated cities [19]. Air pollution, especially PM_2.5_ exposure, is associated with the increased lung cancer risk and mortality independent of cigarette smoking [20]. It is difficult to pinpoint the carcinogenic role played by single constituent of air pollution. Significant associations are found with specific PM components such as PM_2.5_ and lung cancer [21]. PM_2.5_ concentration plays a dominant role in inducing lung cancer, which is consistent with the evidence that lung cancer incidence without tobacco smoking history is increasing in some large cities with air pollution problem [22, 23]. The increased risk estimates of lung cancer are observed for each 10μg/m^3^ increment in PM_2.5_ concentration [24]. There is an excess risk of approximately 19% for lung cancer per 10 mg/m^3^ increment in the long-term average exposure to fine particulates [25]. A meta-analysis finds significant association between risk for lung cancer and PM_2.5_ (HR 1.18 per 5 mg/m^3^) [26].

Chronic Diseases and metabolic disorders affect lung cancer mortality. Chronic lung Diseases, such as COPD, Alpha1-antitrypsin deficiency carriers, patients with interstitial fibrosis or idiopathic pulmonary fibrosis, have shown strong association with lung cancer [27–30]. Cancer-related lung cancer death is higher among people with diabetes [31]. Compared with normal weight, the relative risk for lung cancer is 0.77 for excess body weight with a BMI of 25 kg/m^2^ or greater [32], while underweight is associated with lower lung cancer risk in a nonlinear, inverted U-shaped relationship [33]. Waist circumference has been found to be positively associated with lung cancer risk in smokers [34].

Genetic components, relating to host susceptibility to cigarette smoke exposure and to certain types of lung cancer development or to an individual responsiveness to therapies, are risk factors of lung cancer pathogenesis. The importance of lung cancer family history with early onset of lung cancer family members, has been highlighted by the incorporation into several lung cancer risk prediction algorithms [35, 36]. The findings on familial aggregation of lung cancer are consistent with a two-fold increase risk for lung cancer in smokers with a family history of lung cancer and also with an increased risk present in non-smokers [37]. Large-effect genome-wide associations for SCC with the rare variants BRCA2 and CHEK2 has been described [38]. The direct evidence is also provided by the increased risk of lung cancer associated with rare Mendelian cancer syndromes in carriers of constitutional TP53, retinoblastoma gene mutations, xeroderma pigmentosum, Bloom’s and Werner’s syndromes [39–43].

**Fig. 1:**
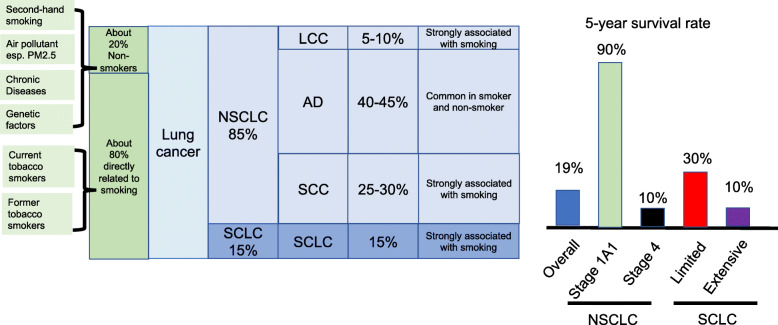
Distribution of cases in lung cancer, 5-year survival rate of lung cancer based on subtypes and stages, and risk factors contributing to lung carcinogenesis. Small-cell lung carcinoma (SCLC) and non-small cell lung carcinoma (NSCLC), accounting for 15% and 85% of all lung cancers respectively. Squamous cell carcinoma (SCC), adenocarcinoma (AD), and large cell carcinoma (LCC) comprises 25–30%, 40-45% and 5–10% of lung cancers. The overall 5-year survival rate of lung cancer is about 19%. Low lung cancer survival rates are due to patients diagnosed with metastatic disease. Among patients with NSCLC, 5-year relative survival rate reach 90% for stage 1A1 but drop below 10% for stage 4. Among patients with SCLC, 5-year relative survival rates are about 30% for limited disease and below 10% for extensive disease. About 80% of lung cancer cases are directly related to smoking. About 20% of lung cancer cases are developed in Non-smokers.

Currently, most patients with lung cancer are diagnosed in the advanced stage, which makes the cancer difficult to surgically resect and increases the postoperative recurrence rate. On the other hand, lung cancer treatment is evolving to precision therapy based on changes in molecular and gene levels [44]. The relative information of lung cancer is summarized in Fig. 1. Further investigation on lung cancer carcinogenesis, diagnosis, therapy, and prognosis will significantly improve the overall survival of patients with lung cancer.

### Exosome and its biological properties and functions

**Fig. 2:**
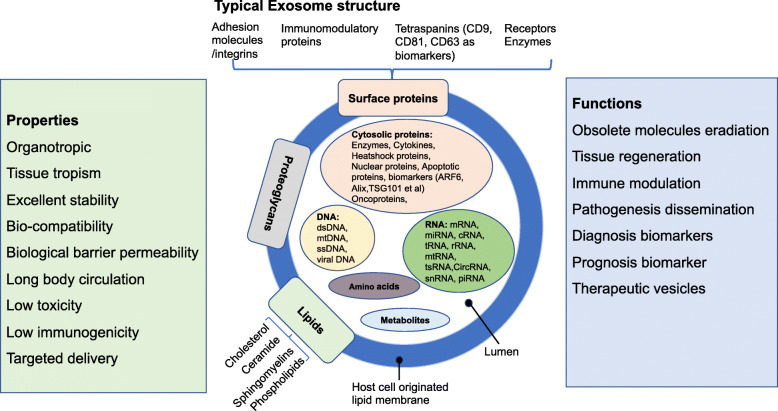
Typical structure, biological properties and functions of exosomes. Exosomes (size range ~40 to 160 nm) are extracellular vesicles generated by all cells. Exosomes can contain different types of cell surface proteins, intracellular protein, RNA, DNA, amino acids, and metabolites. Exosome surface proteins include tetraspanins, integrins, immunomodulatory proteins, and more. Several molecules are used as exosome markers (CD9, CD81, CD63, TSG101, ceramide, and Alix). Exosomes can be a highly heterogeneous population with pleiotropic functions. They are mediators of near and long-distance intercellular communication in health and disease and affect various aspects of cell biology.

Exosomes are endosomal origin and nano-sized vesicles with a size range of ~40-160 nm (average ~100 nm) in diameter [10]. All cells release extracellular vesicles (EVs), which are broadly divided into ectosomes and exosomes. Ectosomes (size range of ~50nm-1mm in diameter) pinch off the surface of the plasma membrane via outward budding. Exosomes are endosomal origin and nano-sized vesicles with a size range of ~40-160 nm (average ~100 nm) in diameter. Hence, two defining distinctions between ectosomes and exosomes include the site of biogenesis and particle size. More and more studies have focused on exosomes because of their crucial roles in cellular homeostasis maintenance and cell communication under pathological or physiological conditions, in exosome-based non-invasive liquid biopsy diagnosis, and in their application of drug delivery [10]. Exosomes are enclosed by a lipid bilayer and contain many cell constituents, including cytosolic and cell surface proteins, glycans, lipids, metabolites, amino acids, RNA, and DNA [11]. The structure and contents of exosomes are demonstrated in Fig. 2. So far, 9769 proteins, 3408 mRNA, 2838 miRNAs and 1116 lipids in various exosomes from different types of cells have been discerned [45].

Surrounding by a host-cell-origin lipid membrane, exosome exhibits intrinsic organotropic and displays tissue tropism mediated by surface molecules, such as integrins and glycans [46]. Exosomal tetraspanin complexes as constitutive components are also involved in the target cell selection [47]. Exosomes possess properties of excellent stability, biocompatibility, biological barrier permeability, long body circulations, low toxicity, low immunogenicity, and unique surface protein expression originated from their parental cells, all of which are good for their target delivery [48]. Because of these properties, exosomes play significant roles in various biological functions and regulate numerous physiological and pathological processes in various diseases. Also, exosomes are promising biomarkers for diagnosis/prognosis of various diseases, which may contribute to the development of minimally invasive diagnosis and next generation therapies. Exosome functions depend on their ability to interact with recipient cells and deliver their contents to these cells [49]. Exosomes are involved in obsolete molecules eradication [50]; antigen presentation [51]; tumor progression, by promoting angiogenesis and tumor cell growth and migration [11], regulatory T lymphocytes differentiation or myeloid cells suppressing immune responses [52]; and pathogenesis dissemination through interaction with recipient cells [53]. The biocompatible nature of exosomes could enhance the stability and efficacy of imaging probes and therapeutics [54]. Due to their potential use in clinical applications, exosomes have attracted much research attention on their roles in health and disease. The biological properties and functions of exosome are summarized in Fig. 2.

### Exosome biogenesis and cellular uptake

Exosome biogenesis originates in the endocytic pathway [10, 11]. It begins with invagination of endosomal limiting membranes by formation of the early sorting endosomes (ESEs). Some of ESEs therefore contain membrane and luminal constituents, which can represent diverse origins. ESEs give rise to late sorting endosomes (LSEs). Second invagination in the LSE leads to generation of intraluminal vesicles (ILVs). This step can lead to further modification of cargoes in the future exosomes, and ultimately form multivesicular bodies (MVBs). Some MVBs end up in lysosomes with enzymes to degrade the components of MVBs. However, some MVBs escape degradation due to the presence of specific surface proteins, such as tetraspanins and lysosomal-associated membrane proteins LAMP1 and LAMP2. These MVBs then fuse with the plasma membrane and get released as vesicles termed as exosomes [55]. Exosome formation requires the coordinated efforts of several protein networks in cell: Rab GTPase proteins control endosomal trafficking [56]; endosomal sorting complexes required for transport (ESCRT), which consists of multiple protein complexes, regulate ILV formation [57]; tetraspanins as transmembrane proteins induce membrane curvatures to enable vesicle formation [58]; and various lipid-modifying enzymes (such as sphingomyelinase, which generates ceramides) promote vesicle formation [59]. TSG101 (tumor susceptibility gene 101) [60], ALIX (apoptosis-linked gene 2-interacting protein X)/Syntenin/syndecan complex [60, 61], phospholipids [62], Flotillin [63], ARF6 and SNARE complex proteins [64] are also involved in the origin and biogenesis process of exosomes, although their functions in exosome biogenesis require further exploration. These factors interact directly with exosomal cargoes and are tightly coupled with the destined substrates for exosome secretion.

Several mechanisms and pathways have been associated with exosome uptake [65, 66], Exosome fusion with cellular membrane of recipient cell lead to the release of exosomal cargoes into cytoplasm. Human melanoma cells uptake exosomal cargoes through exosome fusion with the plasma membrane [67]. Juxtracrine signaling through receptor-ligand interactions is also involved in cellular uptake of exosome. There are several proteins that may act as potential receptors for exosome uptake, such as Tim1/4 for B cells [68] and ICAM-1 for APCs [69]. Various types of endocytosis have been identified as possible mechanisms of cellular uptake of exosome. Endocytosis by phagocytosis and micropinocytosis are much more common method of exosome uptake [70, 71]. Clathrin-dependent endocytosis [72, 73], lipid raft dependent [74], and caveolae endocytosis [64] are the other three kinds of endocytosis for exosome uptake.

**Fig. 3:**
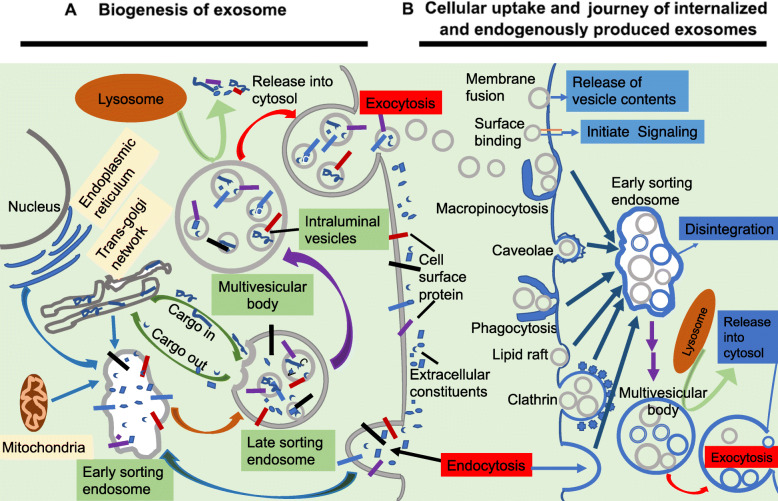
Biogenesis of exosomes, and cellular uptake and journey of internalized and endogenously produced exosomes. **a** Biogenesis of exosomes. Extracellular constituents along with cell surface proteins enter cells through endocytosis of plasma membrane segments. This resulting plasma membrane bud leads to the formation of Early sorting endosomes (ESEs) or fuses with ESEs pre-formed by the constituents of the endoplasmic reticulum (ER), trans-Golgi network (TGN), and mitochondria. ESEs give rise to Late sorting endosomes (LSEs). Invagination in the LSE results in the Multivesicular bodys (MVBs) generation containing Intraluminal vesicles (ILVs) as future exosomes. MVBs can ultimately undergo degradation in the lysosomes with the degradation products recycled by the cells. MVBs can also be transported to the plasma membrane and dock on the luminal side of the plasma membrane. Exocytosis follows and results in the release of the exosomes. **b** Cellular uptake and journey of internalized and endogenously produced exosomes. In the recipient cell (which can be the exosome-producing cell itself), exogenous exosomes may be internalized by multiple pathways and can undergo various fates. Depending on the cell type, extracellular exosomes can dock either at the plasma membrane to release their intraluminal contents into the cytoplasm of recipient cell by fusion. They can remain bound to the surface (for example, to integrins) and initiate intracellular signalling pathways (for example, antigen presentation). Extracellular exosomes may also be internalized by various types of endocytosis: phagocytosis, micropinocytosis, clathrin-dependent endocytosis, lipid raft dependent endocytosis, and caveolae endocytosis. Internalization will target exogenous extracellular exosomes into the canonical endosomal pathway, whereby they reach MVBs, in which the internalized exosomes are likely to mix with endogenous ILVs. Exosome biogenesis and cellular uptake pathways intersection results in a composition of net production of mixed endogenous and exogenous exosomes population.

In any given cell, exosome biogenesis and cellular uptake pathways may intersect, resulting in a composition of net production of mixed endogenous and exogenous exosomes population (Fig. 3).

### Exosome isolation and characterization

Generally, exosomes can be isolated from a variety of body fluids and conditioned cell culture media, such as blood, semen, saliva, plasma, urine, cerebrospinal fluid, epididymal fluid, amniotic fluid, malignant and pleural effusions of ascites, bronchoalveolar lavage fluid, synovial fluid, and breast milk [75, 76]. Several conventional methods have been employed to isolate exosomes as: 1) Ultracentrifugation, including differential and density-gradient ultracentrifugation, is conventional method and suitable for pelleting lipoproteins, extravesicular protein complexes, aggregates, and other contaminants [77]. In addition, density-gradient ultracentrifugation, which separates particles by layers of biocompatible medium with varying densities (e.g., sucrose), yield the exosome preparations with a higher purity as compared to a classic ultracentrifugation and gain great popularity in the currently used exosome strategies [78]. However, it cannot apply for small volumes of clinical samples and is time-consuming, labor-intensive, costly instrumentation requirement, and multiple overnight centrifugation steps included [77]; 2) Ultrafiltration (UF), including Size-Based Filtration, Size-Exclusion Chromatography, and Polymer Precipitation, is size-based exosome isolation method using membrane filters with defined molecular weight or size exclusion limits [79]. UF is faster than ultracentrifugation, and does not require costly equipment. The method is not suitable for exosome enrichment, and disadvantages include that different size exosomes are mixed together and non-exosome material such as protein aggregates exist [80]; 3) Immune affinity capture is applied for isolating exosomes based on specific interactions between proteins (antigens) and their antibodies or between receptors and their ligands [81]. It has been used to capture specific exosomes from a complex population, with the advantage of specificity and disadvantage of low yields; 4) Commercially available kits have been emerged to enrich exosomes, such as ExoQuick kit (System Bioscience), Total Exosome Isolation kit (Invitrogen), qEV columns (Izon), the exoEasy Maxi kit (Qiagen), Ultrafiltration (UF) Amicon® ultra-0.5 centrifugal filter devices (Millipore) [82]; 5) Microfluidics-Based Isolation Techniques are essential and important techniques for the microscale isolation, detection, and analysis of exosomes. By using both physical and biochemical properties of exosomes, this technology meets the challenge of providing high-purity exosomes for clinical settings [83]. The device with a size-based exosome isolation chip facilitates high-yield and high-purity exosome isolation from biofluids and clinical samples, including plasma, urine, and lavage, demonstrating its broad applicability to cancers and other diseases [84]. It is reported that construction of anti-CD9 antibody-coupled highly porous monolithic silica microtips allows automated, rapid and reproducible exosome extraction from multiple clinical samples [85]. A microfluidic chip for immunocapture and quantification of EpCAM-positive and HER2-positive exosomes has been developed [86]. Each approach has its advantages and disadvantages, and may be dictated by the sample source and intended exosome uses. Improvements and innovations in isolation methods are essential for translating exosome research into clinical applications and patient care.

**Fig. 4:**
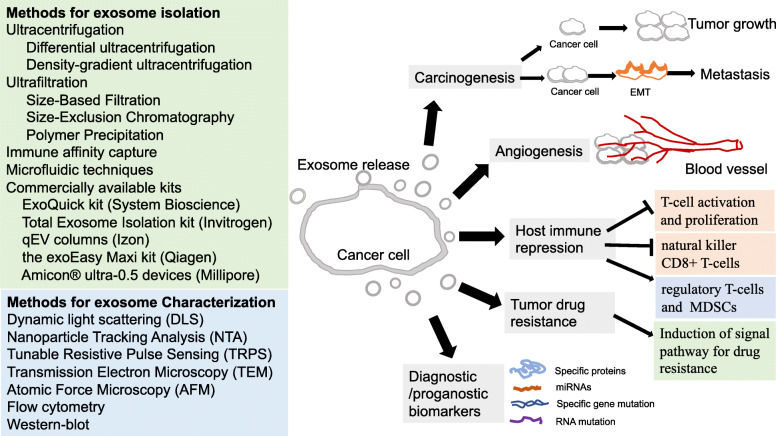
Exosome isolation, characterization, and tumor-derived exosomes role in tumor development. Methods for exosome isolation and characterization are listed. Tumor-derived exosomes role in tumor development includes to promote cancer carcinogenesis, angiogenesis, and drug resistance, help cancer cell escape from host immune system, and can be useful diagnostic and/or prognostic biomarkers.

Exosomes are characterized by size, morphology, flotation density, and presence of marker proteins [10, 11]. Several techniques have been routinely used to characterize exosomes. These include Dynamic light scattering (DLS) [87], Nanoparticle Tracking Analysis (NTA) [87], Tunable Resistive Pulse Sensing (TRPS) [88], Transmission Electron Microscopy (TEM) [89], Atomic Force Microscopy (AFM) [90], flow cytometry [91], and Western blot for detecting marker proteins [10, 11]. Each of these techniques has its own limitations that must be taken into consideration: 1) DLS is an alternative technique for measuring exosome size to provide diameter range of the analyzed vesicles, while does not provide any biochemical data [87]; 2) NTA can measure the exosome movement by tracking each particle through image analysis and then to correlate this movement to particle size [87]. Advantage of using NTA includes quick sample preparation, easy and quick measurement, and samples recovery to their native form after NTA measurements. Moreover, the presence of antigens on exosomes can be detected by applying fluorescent-labeled antibodies [87, 92]; 3) TRPS, with the salient feature of in situ single-particle characterization and concentration measurement, is the most useful for measuring exosomes size distribution and concentration, characterizing particles ranging from approximately 50 nm in diameter up to the size of cells, and investigating cellular function and uptake [88]. Essentially, TRPS measures the brief increase in electrical resistance produced by individual nanoscale particles as they translocate through a size-tunable micrometer-sized pore. The length of the resistive pulse can be correlated to the particle size. In addition, the number of resistive pulses over a given time reveals particle concentration within a sample [88]. Its defects include system stability issues due to the blockage by particles, and sensitivity issues due to too small particles to be detected against the system background noise [88]; 4) TEM is a widely used technique to characterize the structure, morphology, and size of various biological components [89]. Consideration should be given on sample preparation, which is extensive, involves multiple steps, and may induce changes in exosome morphology. Moreover, in some cases, the electron beam may also damage biological samples. Frozen exosomes examined by cryo-TEM is an alternative method which has been shown to well maintain exosome morphology [49]; 5) AFM is a unique technique for studying exosomes, which measures samples in native conditions with minimal sample preparation and without any destructive operation mode [90]. The defect is that sample characterization carried out from external analyses lead to different experimental conditions affecting the measure [90]; 6) Flow cytometry is a molecular approach and one of the most frequently used techniques to characterize exosome size, structure, and surface proteins [91]. It is well-adapted to the reproducible analysis of clinical samples, allowing measurement of exosomes size and structure [91]. 7) Western blot for detecting marker proteins is also used for exosome confirmation (Fig. 1 and Fig. 4) [10, 11]. It is the most commonly used methods to quantify proteins in exosomes. Common proteins that can identify exosomes must be analyzed to demonstrate the nature, purity degree and functional activities of exosome preparation. Furthermore, several proteins that are frequently enriched in specific cancer (eg., lung) cell-derived exosomes should be considered for screening cancer biomarkers [93, 94]. The various methods of exosome isolation and routinely used techniques of exosome characterization have been summarized in Fig. 4.

### Tumor-derived exosomes

Secreted by cancer cells, Tumor-derived exosomes (TDEs) might modulate the recipient cells activity and play important roles in tumor growth and metastasis through participating in cellular communications, modulating cell signaling, and contributing to pre-metastatic niche (PMN) formation [11, 95–97]. Recent evidences indicate that TDEs have significant effect on of cancer angiogenesis by transferring proteins such as VEGF, FGF, IL-6, IL-8 and angiopoietin [98]. TDEs modulate antitumor immune responses by inhibiting T-cell activation and proliferation, inducing regulatory T-cells and myeloid-derived suppressor cells (MDSCs), and inhibiting natural killer (NK) and CD8+ T-cells function, thus to facilitate tumor progression by evasion of host immune system [99–101]. Furthermore, TDEs from drug-resistant cancer cells incorporate cell surface P-glycoprotein from their donor cells. They effectively bind to drug-sensitive recipient cells and transfer functional P-glycoprotein to the latter, which are crucial importance in induction of signal pathway for drug resistance in recipient cells [102]. With a significantly increased number traced in cancer patient blood than in normal human blood, TDEs can be useful diagnostic and prognostic biomarkers [103]. Collectively, TDEs can promote cancer carcinogenesis, angiogenesis, and drug resistance, help cancer cell escape from host immune system, and can be useful diagnostic and/or prognostic biomarkers (Fig. 4).

### Exosome in lung cancer

**Fig. 5:**
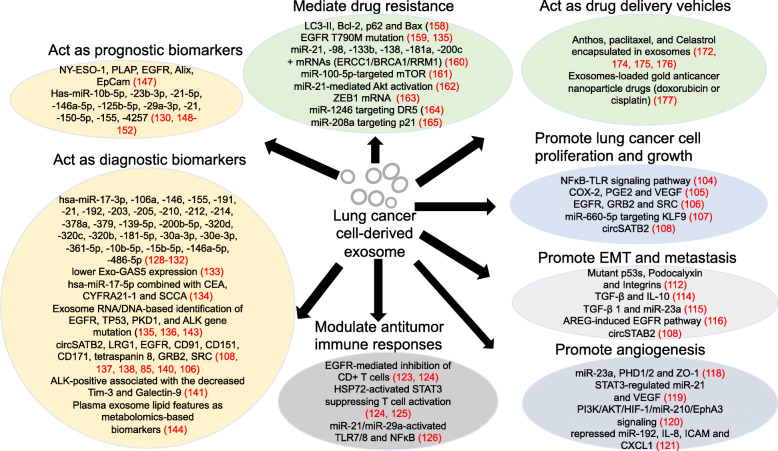
Summary of the potential molecules in lung cancer cell-derived exosomes in carcinogenesis, diagnosis, prognosis, and therapy of lung cancer. Lung cancer cell derived-exosomes have potential role in promoting lung cancer growth, metastasis, and angiogenesis; modulating antitumor immune responses and drug resistance to lung cancer; acting as diagnosis and prognosis biomarkers in lung cancer; and exploring as therapy vesicles for lung cancer drugs.

The poor survival rates of lung cancer are mainly due to late-stage diagnosis and/or inefficient therapeutic regimens. Lung cancer cell derived-exosomes (LCCDEs) have received recent interest in their pivotal role in lung cancer carcinogenesis, early detection/diagnosis, drug resistance, exosome drug delivery system establishment for target therapy, and prognosis, which are summarized in Table 1 and Fig. 5.

**Table 1:** Exosomes and exosomal molecules involved in lung cancer carcinogenesis, diagnosis, prognosis and therapy

Exosome promotes lung cancer cell proliferation and growth						
2003Exosome/Molecule	Parental cell	Recipient cell	Target(s) /mechanisms	Functions		Reference
A549-derived exosomes	NSCLC cell A549	Mesenchymal stem cells	NFκB-TLR signaling pathway	Promote MSCs getting tumor supportive characteristics		[104]
COX-2	lung cancer cells H460,11-18	Monocytic cell THP-1	PGE2 and VEGF	Affect the microenvironments		[105]
EGFR, GRB2 and SRC	NSCLC cells A549, HCC827	HBE3(bronchial epithelial cells)		Actively regulate recipient cells proliferation		[106]
miR-660-5p	NSCLC cells H1299, H460, A549, H358; 16HBE (bronchial epithelial cells)		KLF9	Promote NSCLC progression		[107]
circSATB2	H1299 cells transfected with circSATB2-OE or circSATB2-sh	H460, A549, BEAS-2B	FSCN1	Promote NSCLC cells proliferation and metastasis, induce proliferation in normal human bronchial epithelial cells.		[108]
Exosome promotes EMT and metastasis						
Exosome/Molecule	Parental cell	Recipient cell	Target(s) /mechanisms	Functions		Reference
Mutant p53s, podocalyxin	H1299 (p53R273H /R175H)	H1299 (p53−/− )	Integrin trafficking	Drive integrin trafficking and cell migration		[112]
TGF-β and IL-10	SCLC cell H1688, NSCLC cell H2228	HMEC-1		Regulate the cellular migration of tumor cells		[114]
miR-23a	NSCLC cell A549		miR-23a significantly enriched by TGF-β1	Be involved in the EMT		[115]
AREG	NSCLC cells CRL-2868, A549	Murine macrophage cells Raw 264.7	EGFR pathway	Trigger a vicious cycle in osteolytic bone metastasis		[116]
circSATB2	H1299 cells transfected with circSATB2-OE or circSATB2-sh	H460, A549, BEAS-2B	FSCN1	Induce cell proliferation, and promote NSCLC cells metastasis		[108]
Exosome promotes angiogenesis						
Exosome/Molecule	Parental cell	Recipient cell	Target(s) /mechanisms	Functions		Reference
miR-23a	Lung adenocarcinoma cell CL1-5	HUVEC (Umbilical vein endothelial cells)	PHD1, PHD2 and ZO-1	Induce angiogenesis and increase permeability in HUVEC.		[118]
miR-21	Cigarette smoke extract (CSE)-transformed HBE cells	HBE, HUVEC	STAT3 activation, VEGF	Increase VEGF levels in recipient cells to promote angiogenesis and malignant transformation.		[119]
miR-210	Tagged A549L cells	HUVEC	PI3K/AKT/HIF-1/miR-210/EphA3 signaling	Promote tube formation activity in HUVECs and increase angiogenesis in A549L-derived tumor xenografts.		[120]
miR-192	NSCLC cell A549 and metastatic subpopulations of A549 M1/M3 /M4	HUVEC	IL-8, ICAM and CXCL1	Induce angiogenesis and associate with high metastatic activity of bone due to a repressed miR-192 regulation on IL-8, ICAM and CXCL1.		[121]
Exosome modulates antitumor immune responses						
Exosome/Molecule	Parental cell	Recipient cell	Target(s) /mechanisms	Functions		Reference
EGFR	30 NSCLC patients’ lung biopsies for exosomes extraction and then pool together	Dendritic cells (DC) CD3+ CD4+ CD25− Th0 cells CD8+ T cells	Induce DCs into tolerogenic DCs to facilitate formation of Tregs and inhibit CD8+ T cells anti-tumor function	Help immune escape of lung cancer cells and promote lung cancer growth.		[123, 124]
Hsp72	Lung adenocarcinoma cells H23	MDSCs (myeloid-derived suppressor cells)	Hsp72/TLR2 induced Stat3 activation	Suppress T cell activation		[124, 125]
miR-21 miR-29a	Human A549, SK-MES and murine LLC cells, Dotap liposomal formulations of interest miRNAs (mimicking the exosomes)	Macrophages and spleen cells from WT and TLR7^−/−^ B6 mice TLR7-/TLR8- human HEK-293 cells WT and TLR7^− /−^ B6 mice	Activate TLR7 and TLR8 on immune cells	Activate NFκB and prometastatic inflammatory response, ultimately leading to tumor growth and metastasis		[126]
Exosomal contents as potential diagnostic biomarkers in lung cancer						
Exosomal contents	Type of lung cancer studied	Number of patients/cells studied	Biopsy material	Method for exosome extraction	Potential roles in clinical practice	Reference
miR-17-3p miR-21 miR-106a miR-146 miR-155 miR-191 miR-192 miR-203 miR-205 miR-210 miR-212 miR-214	Adenocarcinoma	36 (27patients + 9 healthy controls)	Plasma	Exclusion chromatography and magnetic activated cell sorting using anti-epithelial cell adhesion molecule	Might be useful as a screening test for lung adenocarcinoma.	[128]
miR-378a miR-379 miR-139-5p miR-200b-5p	Adenocarcinomas + carcinomas	105 (50 lung adenocarcinomas +30 granulomas +25 healthy smokers)	Plasma	ExoQuick	As potential diagnostic biomarkers of lung adenocarcinoma.	[129]
miR-320d miR-320c miR-320b	Patients with advanced EGFR/ALK WT NSCLC who received PD-1/PD-L1 inhibitors	37 (30 patients+ 7 healthy individuals)	Plasma	Ultracentrifugation	As potential biomarkers for predicting the efficacy of immunotherapy in advanced NSCLCs.	[130]
miR-181-5p miR-30a-3p miR-30e-3p miR-361-5p miR-10b-5p miR-15b-5p miR-320b	Early-stage NSCLC patients	88 for miRNA-seq (46 stage I NSCLCs + 42 healthy controls) 60 symptomatic patients for RT-PCR	Plasma	Ultracentrifugation and Immunoaffinity magnetic beads	AD-specific miR-181-5p/-30a-3p, /-30e-3p/-361-5p SCC-specific miR-10b-5p/-15b-5p/-320b as effective biomarkers for early NSCLC diagnosis.	[131]
miR-146a-5p miR-486-5p	Early NSCLC at stages I/II	128 (48 NSCLC patients at stages I/II + 32 patients with lung benign lesion + 48 healthy controls	Serum	Exosome extraction kit (Umibio Shanghai)	Serum exosomal miRNAs other than serum miRNAs be preferable biomarkers for NSCLC at early stages.	[132]
GAS5	NSCLC	104 (64 NSCLC + 40 healthy subjects)	Serum	Exosome Isolation Kit (from serum; Thermo, Carlsbad, CA)	Lower expression of Exo-GAS5 may be a marker for early-stage NSCLC.	[133]
miR-17-5p CEA CYFRA21-1 SCCA	NSCLC	190 training set (100 NSCLC patients + 90 healthy controls) 119 validation set (72 NSCLC patients + 47 healthy controls).	Serum	ExoQuick™	Have clinical value in diagnosis of NSCLC.	[134]
circSATB2	NSCLC	178 (83 NSCLC patients + 95 non-cancerous donors)	Serum	Ultracentrifugation	Be potential biomarker for diagnosis of lung cancer and cancer metastasis.	[108]
EGFR mutations	NSCLC	84 patients enrolled in TIGER-X (NCT01526928)	Plasma	ExoLution^TM^ Plus Extraction technology (Exosome Diagnostics, Inc.)	Combining exoRNA and ctDNA increases the sensitivity for EGFR mutation detection.	[135]
exoDNA	A broader panel of cancer cell lines, including lung, melanoma, breast, prostate and pancreatic cancers.	lung, melanoma, breast, prostate and pancreatic cancer cell lines + two normal stromal fibroblast lines	Cell culture medium	Differential ultracentrifugation	Highlight the value of exoDNA in TDEs as biomarkers in the early detection of cancer and metastasis.	[136]
LRG1	NSCLC	18 (8 NSCLC patients + 10 healthy controls)	Urine	Ultracentrifugation	Be a biomarker for diagnosis of NSCLC.	[137]
EGFR	Lung cancer patients	9 lung cancer patients + 9 normal controls	Plasma	A targeted ELISA with an anti-CD81 antibody	Be a possible biomarker for characterization of lung cancer.	[138]
CD91	Lung cancer patients	259 (165 lung cancer patients + 29 interstitial pneumonia patients + 64 normal controls)	Serum	Anti-CD9 MSIA tips	May be a lung adenocarcinoma biomarker.	[85]
CD151 CD171 tetraspanin 8	Lung cancer patients	581 (431 lung cancer patients + 150 controls)	Plasma	extracellular vesicle array contained 49 antibodies for capturing exosomes	Be promising diagnostic biomarkers in lung cancer	[140]
EGFR GRB2 SRC	NSCLC cell lines	NSCLC cells A549, HCC827 + Bronchial epithelial cells HBE3, HBE4	Cell culture medium	Ultracentrifugation	May be as biomarkers for NSCLC patients.	[106]
Tim-3 Galectin-9	NSCLC	159 (103 NSCLC patients including 60 early stages and 43 advanced stages disease samples + 56 healthy subjects)	Plasma	Exosome Precipitation Solution (3D Medicines biotechnology, Shanghai, China)	A decreased Tim-3 and Galectin-9 levels are biomarkers for response to first generation ALK-TKIs.	[141]
TP53 EGFR PKD1 ALK	Lung adenocarcinoma patients	20 stage IV lung adenocarcinoma patients	Plasma Pleural effusion	Ultracentrifugation	As the top 4 mutated genes in plasma-derived exoDNA and pleural effusion-derived exoDNA, may be used in clinical genetic testing.	[143]
exosomal lipids	NSCLC	120 (91 NSCLC subjects (44 early/47 late stage) + 39 normal controls)	Plasma	Ultracentrifugation	Exosomal lipid features as metabolomics-based biomarkers distinguish early stage lung cancer from healthy individuals.	[144]
Exosomal contents as potential prognostic biomarkers in lung cancer						
Exosomal Biomarkers	Type of lung cancer studied	Number of patients/cells studied	Biopsy material	Method for exosome extraction	Roles in clinical practice	Reference
NY-ESO-1 EGFR PLAP EpCam Alix	NSCLC	276 NSCLC patients	Plasma	EV Array slides with biotinylated antibodies (anti-human-CD9, -CD63 and -CD81)	Act as strong prognostic biomarker in NSCLC.	[147]
miR-10b-5p miR-23b-3p miR-21-5p	Adenocarcinoma	20 (10 adenocarcinoma patients + 10 healthy controls)	Plasma	ExoQuick™	Be promising prognostic biomarkers of NSCLC.	[148]
miR-146a-5p	NSCLC	100 advanced NSCLC patients who received standard cisplatin-based chemotherapy	Serum	ExoQuick™	Downregulated miR-146a-5p indicates higher recurrence rates and predicts the cisplatin effect.	[149]
miR-125b-5p	Patients with advanced EGFR/ALK WT NSCLC who received PD-1/PD-L1 inhibitors	37 (30 patients+ 7 healthy individuals)	Plasma	Ultracentrifugation	Downregulated miR-125b-5p indicates well respond to immunotherapy in patients.	[130]
miR-29a-3p miR-150-5p	NSCLC Human NSCLC cell lines NCI-H460, A549, NCI-H1299 and embryonic lung fibroblasts MRC5, IMR90	5 patients with Stage IIIA NSCLC under radical thoracic RT for miRNA profiling 21 NSCLC patients receiving radical thoracic RT for validation cohort 5 lung cell lines	Plasma Cell culture medium	Solvent-exchange exosome isolation kit (Exiqon)	Decreased miR-29a-3p and miR-150-5p in exosomes are biomarkers to predict unexpected responses to radiation therapy, such as toxicity.	[150]
miR-21 miR-155	Lung cancer cell NCI-H1299, The established subcutaneous primary and recurrent lung cancer xenografts in nude mouse	Lung cancer cell NCI-H1299 + normal lung cell BEAS-2B + 25 nude mice inoculated with NCI-H1299 cells	Cell culture medium tumor and serum	Ultracentrifugation	Be significantly upregulated in serum exosomes of recurrent tumor-bearing animals.	[151]
miR-21 miR-4257	NSCLC	201 NSCLC patients	Plasma	Ultracentrifugation	Be potential as predictive biomarkers for recurrence in NSCLC patients.	[152]
Exosomes in the targeted drug resistance of lung cancer						
Exosome/Molecule	Parental cell	Recipient cell	Target(s) /mechanisms	Functions		Reference
Exosomes obtained from EGFR-mutant NSCLC cells PC9	PC9 cells treated with gefitinib (for Exo-GF) or cisplatin (for Exo-DDP)	PC9 cells treated with gefitinib or cisplatin	Upregulate LC3-II and Bcl-2 and downregulate p62 and Bax.	Exo-GF increases autophagic activity, leading to cells an increased cisplatin resistance.		[158]
EGFR withT790M mutation	84 patients enrolled in TIGER-X			Lead to gefitinib resistance. A 90% sensitivity for detection of EGFR T790M using exoRNA.		[135, 159]
Exosomes after A549 cells exposure to cisplatin (DDP)	A549 cells exposure to DDP	A549 cells	Increased miR-21, ERCC1/BRCA1/RRM1 mRNAs, and decreased miR-98/-133b/-138/-181a/-200c	Mediate DDP drug resistance via miRNAs and mRNAs exchange by exosomes.		[160]
miR-100-5P	A549/DDP cells (A549 resistance to cisplatin)	A549 cells	miR-100-5p-targeted mTOR	Increase cisplatin resistance of recipient cells.		[161]
miR-21	Lung cancer cells H827R (gefitinib-resistant HCC827)	NSCLC HCC827	miR-21-mediated Akt activation	Mediate the transfer of drug resistance.		[162]
ZEB1 mRNA	Mesenchymal cells HBEC	Bronchial epithelial cells HBECs		Transfer chemoresistance and mesenchymal phenotypes to recipient cells.		[163]
miR-1246	Irradiated A549 and H446 cells	A549 and H446 cells	DR5 gene	Act as a messenger between irradiated and non-irradiated cells and promote cell radioresistance.		[164]
miR-208a	NSCLC cells A549, H1299, H1975, H460 Bronchial epithelial cells BEAS-2B, HBE		Targeting p21 with a corresponding activation of AKT/mTOR pathway	Increases the proliferation and radioresistance of human lung cancer cells.		[165]
Exosomes as drug delivery vehicles in the targeted therapy of lung cancer						
Exosome	Drug loaded	Type of lung cancer treated	Method for exosome extraction	Functions		Reference
exosomes from raw bovine milk	Anthos	A549, H1299 Nude mice bearing A549 xenografts	Ultracentrifugation	Enhance therapeutic response compared with the free Anthos against lung cancer tumor with no signs of gross or systemic toxicity.		[172]
exosomes released by macrophages RAW 264.7	paclitaxel	Murine Lewis lung carcinoma cells 3LL-M27, C57BL/6 mice intra-tail vein injected with 3LL-M27 cells	ExoQuick ™	Reduce the IC50 values of chemotherapeutics and induce antitumor activity.		[174]
milk-derived exosomes	Paclitaxel	Nude mice bearing A549 xenografts	Ultracentrifugation	Show significant tumor growth inhibition of human lung tumor xenografts in nude mice.		[175]
Milk-derived exosomes	Celastrol	NSCLC cells A549, H1299, C57BL/6 mice bearing lung cancer cell xenografts	Ultracentrifugation	Enhance Celastrol efficacy and reduce dose related toxicity.		[176]
Exosomes from H1299 and MRC9 cells ferrying Gold nanoparticles	Doxorubicin cisplatin	NSCLC cells H1299, A549	Ultracentrifugation	Deliver drugs to lung cancer cells and produce therapeutic response with preferential cytotoxicity towards cancer cells.		[177]

### Exosome promotes lung cancer growth and metastasis

LCCDEs can affect lung cancer progression by regulating the physiological functions of surrounding tissue cells and microenvironment. LCCDEs induce mesenchymal stem cell (MSC) transformation into a pro-inflammatory phenotype via NFκB-TLR signaling pathway to promote MSCs getting tumor supportive characteristics [104].The induced COX-2 expression in lung cancer cells is transferred to other cells by exosomes, which leads to an increased PGE2 and VEGF production and affects the microenvironments, including COX-2-involved inflammatory reactions [105]. Proteins associated with signal transduction, including EGFR, GRB2 and SRC, are enriched in NSCLC exosomes and actively regulate recipient cells proliferation [106]. High levels of miR-660-5p in NSCLC exosomes promote NSCLC progression by targeting KLF9 [107]. circSATB2 can be transferred by exosomes and then promotes the NSCLC cells proliferation and metastasis, as well as induces abnormal proliferation in normal human bronchial epithelial cells [108].

Metastasis is a critical phase of tumor progression and major cause of cancer mortality, which is related to a variety of mechanisms and involves multiple steps [11]. Different types of metastatic cancer cells are significantly different in organ tropism, which is highly associated with TDEs integrins [96]. The main sites of lung cancer metastasis are brain, adrenal gland, bone and liver [155]. LCCDEs, as carriers of information transmission, can increase tumor cell invasiveness by promoting formation of the PMN microenvironment [11, 156]. Recently, it has been found that tumor-associated fibroblast behavior including pro-invasive extracellular matrix (ECM) deposition fosters cancer cell migration and invasion [157]. Mutant p53s in NSCLC cells modulate exosomal podocalyxin expression to impact integrin trafficking in fibroblasts and promote deposition of a highly ECM to create a microenvironment supportive of tumor cell metastasis [109]. There is a synergistic interaction between invadopodia biogenesis and exosome secretion with consequent matrix degradation and overall aggressive behavior in promoting cancer cell invasiveness [158]. TGF-β and IL-10 in LCCDEs enhance tumor cells rapid growth and migration [110]. Also, TGF-β1 and miR-23a in LCCDEs are involved in the EMT, which is an important process before the tumor cells metastasis [111]. NSCLC exosomal Amphiregulin (AREG) induce activation of EGFR pathway in pre-osteoclasts to trigger a vicious cycle in osteolytic bone metastasis [112]. It is certainly critical for future work to better dissect how LCCDEs cargoes serve as a novel cellular communication to regulate lung cancer progression, which can help to elucidate the mechanism of lung cancer development.

### Exosome promotes angiogenesis during lung carcinogenesis

Angiogenesis is vital for tumor growth since tumor vessels are the important nutrient sources for tumor cells [159]. Exosomal miR-23a from hypoxic lung cancer cells can increase angiogenesis and vascular permeability [113]. LCCDEs miR-21, regulated by STAT3, enhances VEGF level to promote tumor angiogenesis and induce malignant transformation of bronchial epithelial cells [114]. LCCDEs miR-210 participates in PI3K/AKT/HIF-1/miR-210/EphA3 signaling to promote tumor angiogenesis [115]. A repressed miR-192 in LCCDEs induces angiogenesis and associates with high metastatic activity of bone [116]. All of these indicate that LCCDEs can promote angiogenesis to facilitate lung carcinogenesis.

### Exosome modulates antitumor immune responses during lung carcinogenesis

LCCDEs can affect the immune cells function in the tumor microenvironment. TDE promotes tumor progression by reprogramming immune cell functions and helps immune escape by transferring specific proteins and RNA into the recipient cells [99, 160]. LCCDEs EGFR induce tolerogenic dendritic cells and tumor antigen-specific regulatory T cells (Treg) to inhibit anti-tumor function of CD8+ T cells and thus promote lung cancer growth [117, 118]. LCCDEs HSP72 can activate the Stat3-dependent immunosuppressive effect of MDSCs (myeloid-derived suppressor cells) to suppress T cell activation [118, 119]. LCCDEs miR-21 and miR-29a can activate Toll-like receptors 7 (TLR7) and 8 (TLR8) on immune cells to activate NFκB and prometastatic inflammatory response, ultimately leading to tumor growth and metastasis [120].

### Exosomal contents as diagnostic or prognostic biomarkers of lung cancer

Liquid biopsy, which is based on detection of tumor-related biomarkers from body-related fluids, represents a minimally invasive and more comprehensive option for tumor early detection and investigation. In clinics, tissue collection is the standard practice for lung cancer diagnosis. However, this method is insufficient to present a complete picture of disease state due to the limited amount and times of tissue collection, and frequent tumor heterogeneity. It may not be sufficient to provide the global disease status, especially in the era of precision and personal medicine. Hence, alternative routes of diagnosis are being explored. LCCDEs contain potential biomarkers for lung cancer diagnosis (Table 1 and Fig. 5). The first study of exosomes in lung cancer shows that exosomes can be isolated from pleural effusions of lung cancer patients [161]. Remarkably, some researchers have succeeded in discovering circulating exosomal miRNAs for their diagnostic value in lung cancer. It has been reported that 12 exosomal miRNAs are overexpressed in blood of lung cancer patients compared with control samples (hsa-miR-17-3p/-21/-106a/-146/-155/-191/-192/-203/-205/-210/-212/-214) [121]. Another report has showed that four exosomal miRNAs (hsa-miR-378a/-379/-139-5p/-200b-5p) are putative diagnostic markers in plasma samples of lung adenocarcinoma patients [122]. Exosomal miRNA expression profile is significantly altered in NSCLC patients compared with normal controls, as miR-320d, -320c, and -320b are identified as potential biomarkers for predicting the efficacy of immunotherapy in advanced NSCLCs [123]. Tumor-derived exosome miRNAs (AD-specific miR-181-5p/miR-30a-3p/miR-30e-3p/miR-361-5p, and SCC-specific miR-10b-5p/miR-15b-5p/miR-320b) may be highly sensitive and noninvasive biomarkers for NSCLC early diagnosis [124]. Serum exosome miRNAs (miR-146a-5p/miR-486-5p) might be preferable biomarkers for NSCLC early diagnosis [125]. The lower expression of noncoding RNA growth arrest-specific transcript 5 (GAS5) in circulating exosomes (Exo-GAS5) could be a noninvasive blood-based tumor marker for early-stage NSCLC identification [126]. The expression of exosomal miR-17-5p, in combination with three circulating tumor markers CEA, CYFRA21-1and SCCA, is significantly higher in NSCLC patients than in healthy controls [127]. Overexpressed circSATB2 in serum exosomes is highly sensitive and specific biomarker for NSCLC diagnosis [108]. Exosomal RNA and DNA have been demonstrated in identification of EGFR mutations of lung tumor origin [128, 129]. The leucine-rich a2-glycoprotein (LRG1) is found to be expressed at higher levels both in urinary exosomes and lung tissue of NSCLC patients [130]. Plasma exosome EGFR expression levels are remarkably different between lung cancer patients and normal controls [131]. Serum exosome CD91 is identified as a lung adenocarcinoma specific marker [85]. The Extracellular Vesicle Array (EV Array) to phenotype lung cancer-related proteins in plasma exosomes may be a simple, minimal invasive tool [162], by which exosomal proteins CD151, CD171 and tetraspanin 8 are found to be higher in lung cancer patients of all histological subtypes [132]. NSCLC exosomal proteome has identified the enriched protein cargoes such as EGFR, GRB2 and SRC for lung cancer early detection [106]. ALK-positive NSCLC patients shows a decreased plasma exosome Tim-3 and Galectin-9 levels [133], which are biomarkers for response to first generation ALK-TKIs [163]. Recently, plasma-derived exosome DNA has been successfully used in clinical genetic test with TP53, EGFR, PKD1, and ALK as the top 4 mutated genes in advanced lung adenocarcinoma patients [134]. Consistent differences in the lipid profiles could reflect the altered expression of many lipid metabolic genes evident in lung tumors. Plasma exosome lipid features as metabolomics-based biomarkers successfully distinguish early stage lung cancer patient from healthy individuals [135]. Exosome heterogeneity can also be conceptualized according to its size. Recent studies have shown that larger exosomes and smaller exosomes exhibit different properties and may have different functions [164]. In particular, the size distribution of exosomes derived from humoral source is different between patients and healthy controls, indicating the importance of size-dependent analysis of exosomes [165]. Therefore, using the size distribution of exosomes as a potential index is helpful for the diagnosis of diseases. A simple, rapid and economical method to reflect the size of exosomes has a broad application prospect in the future. These data (Table 1 and Fig. 5) suggest that development of novel exosome biomarkers for lung cancer diagnosis will be important for enhancing patient outcome and survival.

Exosomal components can be used as non-invasive prognostic biomarkers of lung cancer [136]. Exosome membrane-bound proteins NY-ESO-1, PLAP, EGFR, Alix and EpCam are correlated to NSCLC overall survival (OS), indicating these proteins as strong prognostic biomarkers [136]. The elevated levels of exosomal miR-10b-5p, miR-23b-3p and miR-21-5p are associated with poor OS and are potential prognostic biomarkers of NSCLC [137]. Downregulation of serum exosome miR-146a-5p indicates a poor progression free survival (PFS) and predicts the cisplatin effect on NSCLC [138]. NSCLC plasma exosome miR-125b-5p as T-cell suppressor is downregulated during the treatment, indicating the increased T-cell function and well respond to immunotherapy in patients [123]. The decreased expression of miR-29a-3p and miR-150-5p in secreted exosomes are biomarkers that correlates with the delivered radiation therapy dose and eventually help predict unexpected responses to radiation therapy, such as toxicity [139]. miR-21 and miR-155 are significantly upregulated in recurrent tumors compared to primary tumors, which are also upregulated in serum exosomes of recurrent tumor-bearing animals [140]. The increased expression of plasma exosome miR-21 and miR-4257 has potential as a predictive biomarker for recurrence in NSCLC patients [141]. All these previous findings are quite encouraging (Table 1 and Fig. 5), representing a novel and less invasive option for prognosis of lung cancer.

Currently, exploring biomarkers in LCCDEs is still at a pre-clinical phase. Although the investigations show promising results for their application in screening programs or as prognostic/predictive biomarkers, there exhibits hurdle for the clinical translation. The main challenges are lack of reliable cutoffs and great variability among the studies. Moreover, no established tool has been approved for exosome isolation of clinical samples. Finally, large prospective clinical trials are mandatory to provide evidence of their clinical utility. Nonetheless, should these and other issues be resolved, LCCDEs may account for promising lung cancer biomarkers in the near future.

### Exosomes in the targeted drug resistance of lung cancer

With the development of molecular biology and tumor genetics, the targeted therapy has become a hot topic, which can guide therapeutic decision and thus reduce morbidity and mortality. Among more than 300 mutations in lung cancer, only a few of these genes such as EGFR, ALK, c-met, ROS1can promote or drive the lung tumorigenesis [166, 167]. More recently, treatment with the immune checkpoint inhibitors targeting axis such as programmed death protein 1 (PD-1) and its ligand (PD-L1) have revolutionized a large proportion of NSCLC patient management [168]. The EGFR mutations reveal a potential responsiveness of NSCLC to TKIs [169]. Drug resistance is a major hurdle in cancer therapy. Exosomes originating from either tumor cells or cancer associated fibroblasts (CAFs) can mediate resistance to chemotherapy, radiotherapy and targeted therapy [170]. Exosomes are involved in inducing lung cancer treatment resistance to concurrent chemotherapy and TKIs. It has been shown that cisplatin sensitivity was significantly reduced when lung cancer cells were treated with cisplatin combined with exosomes derived from gefitinib-treated lung cancer cells, underlining the importance of exosomes in mediating antagonistic effects of cisplatin and gefitinib therapy [142]. T790M mutation is found in patients treated with EGFR-TKIs which leads to gefitinib resistance [143]. Exosomal RNA is used to detect EGFR T790M and activating EGFR mutations, with the sensitivity of 90% and 98% respectively [128]. It has been found that the exosomes released by A549 cells during cisplatin exposure decrease the sensitivity of non-treated A549 cells to cisplatin through several miRNAs (increased expression level of miR-21 while decreased expression levels of miR-98/133b/138/181a/200c) and mRNAs (increased expression levels of ERCC1/BRCA1/RRM1) exchange by exosomes via cell-to-cell communication [144]. Several molecules in LCCDEs are involved in the targeted drug resistance of lung cancer (Table 1 and Fig. 5). Cisplatin-resistant LCCDEs increase cisplatin resistance of recipient cells in exosome miR-100-5p-dependent manner with mTOR as its potential target [145]. Exosome-mediated gefitinib resistance in lung cancer cells is via delivery of miR-21 to activate Akt [146]. Exosomes derived from mesenchymal and oncogenically transformed lung cells can transfer chemo-resistance and mesenchymal phenotypes to recipient cells via exosomal ZEB1 mRNA [147]. Upregulation of miR-1246 and miR-208a as prognostic biomarker, are associated with radiotherapy resistance and high proliferation of the tumor by targeting DR5 and p21 respectively [148, 149]. Although the detailed mechanism of exosome-mediated drug resistance remains unclear, inhibition of LCCDEs formation and release may present a novel therapeutic strategy for lung cancer. Furthermore, it is possible to identify drug targeted therapy resistance of lung cancer patients via screening specific molecules in LCCDEs.

### Exosomes as drug delivery vehicles in the targeted therapy of lung cancer

Therapeutic Deliverable Exosomes are emerging as promising drug delivery agents because of their natural intercellular communication role, excellent biocompatibility, low immunogenicity, low toxicity, long blood circulation ability, biodegradable characteristics and their ability to cross various biological barriers [48, 54]. Homotypic adhesion molecules on exosome membrane endow exosomes with strong preferential binding to source cells [171]. Tetraspanin CD9 and CD81 on exosome membrane can promote direct membrane fusion between exosomes and cells, allowing the exosomal cargo transportation [172]. CD55 and CD59 on exosome membrane can protect exosome from complement attack and increase its stability in circulation [173]. Moreover, high expression level of CD47 on exosome membrane leads to exosome resistance to phagocytosis by monocytes and macrophages [174, 175].

However, making exosomes with satisfactory cancer-targeting ability and controlling encapsulated drug release is highly challenging. Exosomes are engineered to carry anticancer therapeutics by active (sonication, electroporation, or freeze–thaw cycles) or passive (simple incubation with exosomes) drug-loading approaches [150, 176]. Molecules such as lncRNAs, miRNAs, and siRNAs can also be added through genome engineering of the donor cells, which helps in de novo production and incorporation of these molecules into exosomes [177]. The paclitaxel-loaded exosome drug delivery system reduces the IC50 values of chemotherapeutics and induces antitumor activity in mouse lung cancer models [151]. Drugs (Anthos/paclitaxel/Celastrol) encapsulated in exosomes show enhanced therapeutic effect in lung cancer xenografts nude mice [150, 152, 153]. Another research has also shown that exosomes loaded with gold anticancer nanoparticle drugs (doxorubicin/cisplatin) are able to deliver drugs to lung cancer cells and produce therapeutic response [154].

One biological strategy to equip exosomes with targeting properties involves incorporating targeting peptides or proteins by inducing their expression in exosome donor cells [178]. In addition to ligand-mediated targeting, magnetic drug targeting provides an alternative method to improve the level of therapeutic efficacy [179]. However, the targeting efficiency of a single ligand is not satisfied because of receptor saturation phenomenon [180]. To overcome these limitations, dual ligand-based active targeting strategies are developed to enable the engineered exosomes with ability of both specific accumulation and improved drug release at the target tumor site [181]. Recently, a synthetic exosome not only preserves the intrinsic functionalities of native exosome, but also gains multiple abilities for efficient tumor targeting via dual ligand-mediated endocytosis, a controlled release, and thermal therapy at target tumor sites [182]. Tumor-specific targeting of drug-loaded nanoparticles (NPs) can be implemented by both passive and active strategies. Passive targeting exploits the enhanced permeation and retention (EPR) effect of small size NPs (50−200 nm) through the abnormal tumor vasculature to accumulate into tumor sites [183]. Active targeting of NPs relies on PEGylation and surface modification of targeting ligands to efficiently increase the targeting specificity to tumors [184]. However, the synthetic NPs exhibits rapid clearance by the mononuclear phagocyte system (MPS) during in vivo circulation. Exosome-coated NPs can reduce the MPS uptake with significant advantages in terms of prolonged blood circulation time, optimal biocompatibility, and enhanced targeting effect [174, 182]. TDE-coated PLGA NPs, using microfluidic sonication approach to fabricate biomimetic NPs, show superior homotypic targeting efficacy and immune evasion [185]. Another report shows an exosome-coated PLGA NPs loaded with DOX achieve efficiently targeted chemotherapy of triple-negative breast cancer [186].

Current studies indicate that exosomes are ideal drug delivery vehicles to protect the enclosed drugs from degradation or damage due to their lipid bilayer membrane and nanoscale size. Future applications for exosome drug carriers include both allogeneic and autologous applications, the latter can avoid potential immune responses [187]. Multipotent stem cells (MSCs)-derived exosomes are thought to possess limited immunogenicity due to low expression of co-stimulatory molecules, such as class I major histocompatibility complex (MHC) molecules, making them suitable for allogeneic transplantation [187]. It is unclear whether allogeneic exosome sources have the potential to be streamlined and developed as off-the-shelf products used for future drug delivery applications. Isolation of distinct exosome subpopulations that display favorable transport properties has proved challenging with current techniques. The versatile designer exosome combined with synthetic materials aims to be used in individualized precise cancer therapy in the future. And the research endpoint in experimental diagnostics/prognostics/therapeutics of lung cancer is to be translated into clinical applications. Clinical Trials play the key role on translational application of exosome-based diagnostics/prognostics/therapeutics into clinics. A December 7^th^ 2020 search of https://clinicaltrials.gov/ct2/search shows about 18 studies related to exosomes and lung cancer (Table 2). Notably, although most of the studies are related to development of exosome-based diagnosis/prognosis, one study investigates the therapeutic aspects. A dendritic cell-derived exosome (Dex)-based clinical trial in phase II examines the use of Dex as vaccine to impart immunotherapy in combination with metronomic cyclophosphamide (mCTX) therapy in patients with lung cancer (NCT01159288) (Table 2).

**Table 2:** Current Clinical Trials Based on Exosomes in Lung Cancer.

NCT Number	Title	Status	Conditions	Interventions
NCT03542253	Combined Diagnosis of CT and Exosome in Early Lung Cancer	Unknown status	Early Lung Cancer	Procedure: Surgery
NCT04529915	Multicenter Clinical Research for Early Diagnosis of Lung Cancer Using Blood Plasma Derived Exosome	Active, not recruiting	Lung Cancer	Diagnostic Test: Exosome sampling
NCT01159288	Trial of a Vaccination With Tumor Antigen-loaded Dendritic Cell-derived Exosomes	Completed	NSCLC	Biological: Dex2
NCT03830619	Serum Exosomal Long Noncoding RNAs as Potential Biomarkers for Lung Cancer Diagnosis	Recruiting	Lung Cancer (Diagnosis)	Diagnostic Test: collect samples
NCT04629079	Improving the Early Detection of Lung Cancer by Combining Exosomal Analysis of Hypoxia With Standard of Care Imaging	Recruiting	Lung Cancer	
NCT04315753	Circulating and Imaging Biomarkers to Improve Lung Cancer Management and Early Detection	Recruiting	Lung Cancer	Other: LDCT (Low Dose CT)
NCT04427475	Prediction of Immunotherapeutic Effect of Advanced Non-small Cell Lung Cancer	Recruiting	NSCLC	Drug: pabolizumab Drug: nafulizumab
NCT02869685	Clinical Research for the Consistency Analysis of PD-L1 in Lung Cancer Tissue and Plasma Exosome Before and After Radiotherapy	Unknown status	NSCLC	Radiation: radiotherapy
NCT04323579	Validation of Multiparametric Models and Circulating and Imaging Biomarkers to Improve Lung Cancer EARLY Detection.	Recruiting	Lung Cancer	
NCT03317080	Dynamic Monitoring Circulating Tumor DNA in Surgical Patients With Lung Cancer	Active, not recruiting	Lung Cancer	
NCT02921854	Detection of Circulating Biomarkers of Immunogenic Cell Death	Completed	NSCLC	Other: Blood withdrawal
NCT03108677	Circulating Exosome RNA in Lung Metastases of Primary High-Grade Osteosarcoma	Recruiting	Lung Metastases Osteosarcoma	Other: Blood samples
NCT04182893	Clinical Study of ctDNA and Exosome Combined Detection to Identify Benign and Malignant Pulmonary Nodules	Recruiting	Pulmonary Nodules	Diagnostic Test: ctDNA and Exosome Combined Detection
NCT03228277	Olmutinib Trial in T790M (+) NSCLC Patients Detected by Liquid Biopsy Using BALF Extracellular Vesicular DNA	Completed	NSCLC	Drug: Olmutinib
NCT04499794	The Study of Exosome EML4-ALK Fusion in NSCLC Clinical Diagnosis and Dynamic Monitoring	Recruiting	•Untreated Advanced NSCLC •FISH Identified ALK Fusion Positive or Negative	Drug: ALK inhibitor
NCT03236675	Detection of Either the EML4-ALK Gene Rearrangements or the T790M EGFR Mutation in the Plasma of Advanced NSCLC Patients	Active, not recruiting	Carcinoma, Non- Small-Cell Lung	
NCT01629498	Image-Guided, Intensity-Modulated Photon or Proton Beam Radiation Therapy in Treating Patients With Stage II-IIIB Non-small Cell Lung Cancer	Recruiting	•Recurrent NSCLC •Stage II NSCLC AJCC v7 •Stage IIA NSCLC AJCCv7 •Stage IIB NSCLC AJCCv7 •Stage IIIA NSCLC AJCC v7 •Stage IIIB NSCLC AJCC v7	•Radiation: Image Guided Radiation Therapy •Radiation: Intensity-Modulated Radiation Therapy •Other: Laboratory Biomarker Analysis •Radiation: Photon Beam Radiation Therapy •Radiation: Proton Beam Radiation Therapy •Other: Questionnaire Administration
NCT02662621	Pilot Study With the Aim to Quantify a Stress Protein in the Blood and in the Urine for the Monitoring and Early Diagnosis of Malignant Solid Tumors	Completed	Cancer	•Other: blood samples •Other: Urine samples

## Conclusion and perspectives

LCCDEs participate in the lung cancer progression. With deep investigation of LCCDEs-mediated role and signal pathways, the mechanism of lung carcinogenesis can be further elucidated. The highlights of exosome biomarkers include that they contain stable sources of tumor-derived genetic material; Exosome number is higher in patients than in healthy controls; Exosomal miRNAs can discriminate healthy individual from patients, early stage from advanced stage, and recurrence from non-recurrence; Exosomal RNA and DNA have a higher sensitivity in detection of somatic mutations. Accumulating evidence suggests that exosome biomarker has a prospective use in lung cancer diagnosis and prognosis. Lung cancer is a disease worthy of precise and personalized medical treatment. Due to the unique biological characters of exosomes, delivery of therapeutic agents through exosomes is a novel approach that holds a great future in medicine. We summarize the pivotal role of exosomes in lung carcinogenesis, and highlights the exemplary capacity of exosomes to be used as diagnostic, prognostic, and therapeutic agents in lung cancer (Table 1 and Fig. 5).

However, a number of associated challenges must be resolved before exosome-based diagnosis/prognosis and delivery systems are applied in clinical settings. The first and most important limitation is related to exosome isolation and thorough characterization. The lack of standardized exosome isolation techniques, storage methods, and appropriate quality controls has hampered further translation and clinical-grade exosome production. In addition, bulk and scalable exosomes manufacturing remains a major hurdle for clinical translation and production cost control. The biofluids availability and exosomes abundance in biofluids varies frequently. Hence, a standardized method for exosome isolation is warranted to ensure a consistent and repeatable exosome supply. The exosome preparation should also be of high quality, homogenous in nature, and free from other cellular vesicular contamination. Meanwhile, challenge for development of exosome biomarkers is lack of large prospective studies to provide evidence that exosome liquid biopsy can be a valid alternative to tumor tissue biopsy. The foremost challenge associated with biological-origin exosome therapeutic system is maintenance of good manufacturing practices. Hence, the standardized protocols to ensure consistent production of exosomes will be needed before exosome-based therapy enters clinical practice. Although exosome-mediated therapy as well as diagnosis and prognosis appear promising, more research needs to be done before exosome is incorporated into clinical applications.

## Data Availability

All data generated or analyzed during this study are included in this published article.

## Abbreviations

AD: Adenocarcinoma
AFM: Atomic Force Microscopy
ALIX: Apoptosis-linked gene 2-interacting protein X
AREG: Amphiregulin
CAFs: Cancer associated fibroblasts
Dex: Dendritic cell-derived exosome
DLS: Dynamic light scattering
EPR: The enhanced permeation and retention
ECM: Extracellular matrix
EMT: Epithelial-mesenchymal transition
ESEs: Early sorting endosome
Exo-GAS5: Growth arrest-specific transcript 5 (GAS5) in circulating exosomes
ESCRT: Endosomal sorting complexes required for transport
ETS: Environmental tobacco smoke
EVs: Extracellular vesicles
LCC: Large cell carcinoma
LCCDEs: Lung cancer cell-derived exosomes
LCNS: Lung cancer in never smokers
LRG1: Leucine-rich a2-glycoprotein
LSEs: Late sorting endosome
MDSCs: Myeloid-derived suppressor cells
MHC: Major histocompatibility complex
MSCs: Multipotent stem cells
MPS: Mononuclear phagocyte system
MSC: Mesenchymal stem cell
MVBs: Multivesicular body
NK: Natural killer
NNK: 4-(methylnitrosamino)-1(3-pyridyl)-1-butanone
NSCLC: Non-small cell lung carcinoma
NTA: Nanoparticle Tracking Analysis
NPs: Drug-loaded nanoparticles
OS: Overall survival
PD-1 and PD-L1: Programmed death protein 1 and its ligand
PFS: Progression free survival
PM_2.5_: Particulate matter 2.5
SCC: Squamous cell carcinoma
SCLC: Small-cell lung carcinoma
TDEs: Tumor-derived exosomes
TEM: Transmission Electron Microscopy
TLR7 and TLR8: Toll-like receptors 7 and 8
Treg: Regulatory T cells
TRK-A3: Tyrosine receptor kinase A3
TRPS: Tunable Resistive Pulse Sensing
TSG101: Tumor susceptibility gene 101
UF: Ultrafiltration
